# Embodied sharpness: exploring the slicing gesture in political talk shows

**DOI:** 10.3389/fpsyg.2024.1494192

**Published:** 2025-02-06

**Authors:** Silva H. Ladewig

**Affiliations:** Department of German Philology, University of Göttingen, Göttingen, Germany

**Keywords:** recurrent gestures, slicing gesture, gesture sequences, metapragmatic meaning, kinesthetic experiences of gestures, stance taking in political communication, mediated multimodal communication, gesture stabilization

## Abstract

This paper explores the Slicing gesture within German political talk shows, focusing on its role in recurrent gesture sequences observed in German political talk shows. Through a detailed historical overview of recurrent gestures in political communication and an extension of the repertoire of recurrent gestures in German speakers, the study emphasizes the Slicing gesture’s function in stance-taking and self-presentation. Highlighting its forms and functions, the analysis demonstrates how this gesture enacts sharpness, decisiveness, and clarity. The study integrates embodied and phenomenological perspectives, showing how kinesthetic experiences shape the gesture’s meaning and metapragmatic dimensions. By contributing to the understanding of recurrent gestures as multimodal resources in political discourse, the paper sheds light on the interplay between embodied communication and rhetorical style.

## 1 Introduction

Understanding how speakers encode and express points of view is a central topic in linguistics. According to Stubbs (1996, p. 202), “[a]ll utterances encode such a point of view, and the description of the markers of such points of view and their meanings should therefore be a central topic for linguistics.” Stance-taking is an inherent part of interaction, as we inevitably adopt stances whenever we engage in communication (see Du Bois and Kärkkäinen, 2012). Du Bois (2007, p. 163) defines “stance as a public act by a social actor, achieved dialogically through overt communicative means (language, gesture, and other symbolic forms), through which social actors simultaneously evaluate objects, position subjects (themselves and others), and align with other subjects, with respect to any salient dimension of value in the sociocultural field.” This perspective highlights that stance-taking is not merely a lexical phenomenon but a multifaceted activity involving active engagement by individuals. Consequently, stance is diverse and varied, yet consistently intertwined with the pragmatic and social aspects of human behavior. This understanding underscores the contextual, practical, and interactive nature of stance-taking (see Englebretson, 2007, pp. 2–3).

**Figure 1:**
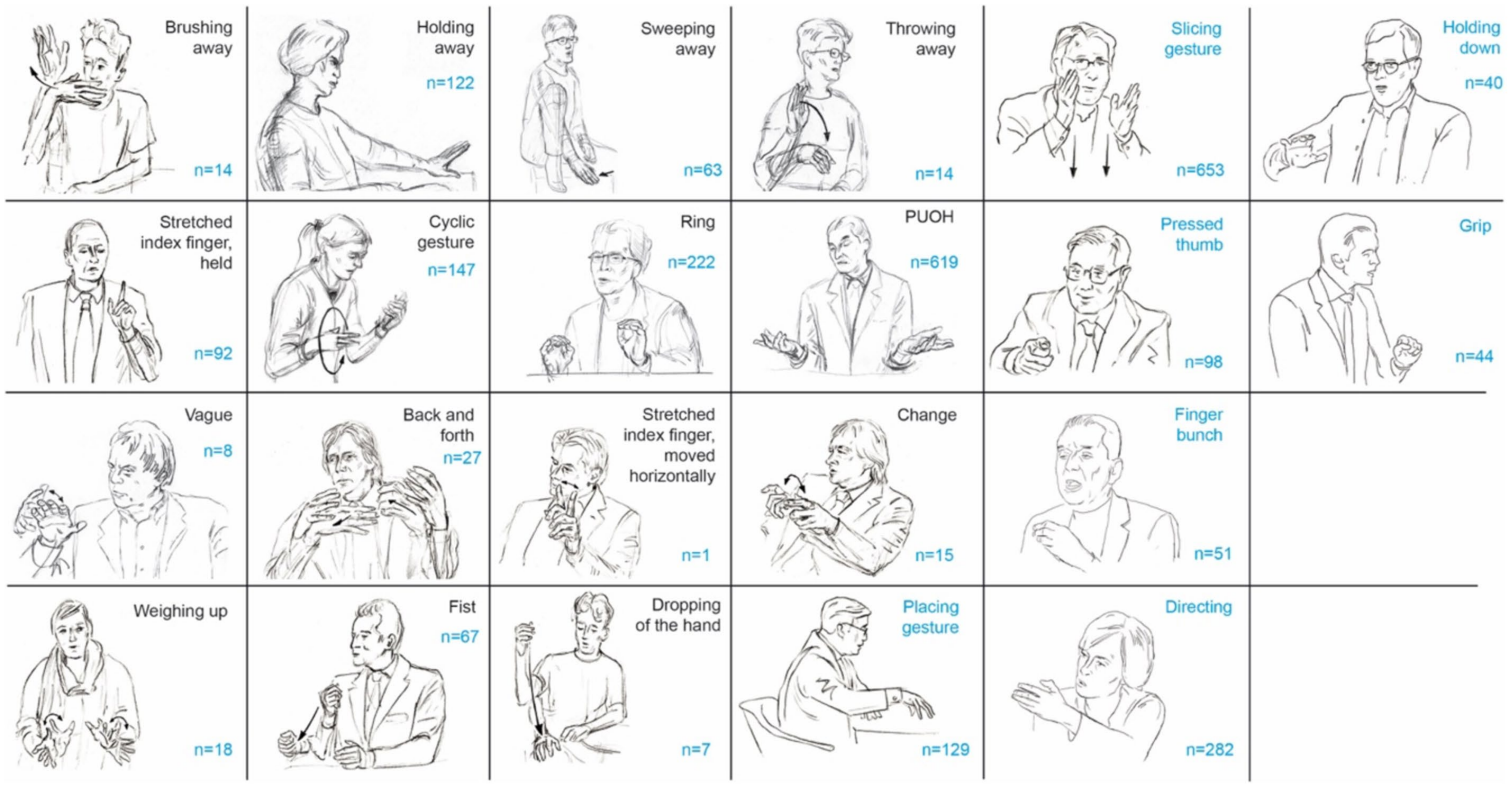
Repertoire of recurrent gestures determined in televised political debates data. The repertoire is based on Bressem and Müller’s (2014) repertoire of recurrent gestures determined for adult German speakers. New candidates, as well as the counts for all identified recurrent gestures in this study, are highlighted in blue.

Bohmann and Ahlers (2022, p. 66) point out that while Du Bois’ definition of stance as a “public act” is broad but generally accepted, its scope leaves the operationalization of stance in specific research contexts somewhat vague. They argue that this ambiguity affects both the formal aspect, which pertains to the nature of the “public act,” and the functional aspect, which involves the variety and interrelationships of different stances taken during interaction. This paper will explore these broad concepts by focusing on how gestures, particularly the recurrent Slicing gesture, characterized by a flat hand with the edge moving downwards, fulfill various functions of stance-taking. By examining the kinesic and functional aspects of gestures in interaction, this study aims to provide a clearer understanding of how different stances are expressed multimodally and related (juxtaposed or contrasted) through gesture sequences. Thus, this paper contributes to the growing body of research on stance-taking, expressed through various embodied means of communication such as gestures, head movements, and gaze (see Andries et al., 2023, for an overview) supporting the position that the concept of stance extends beyond verbal communication. In doing so, the study presented merges two aspects of multimodal, i.e., verbo-gestural, communication: recurrent gesture-based expression of stance and markers of social identity within the context of mediated political debates. Considering the first aspect, gesture studies have demonstrated that recurrent gestures, which are partly conventionalized gestures (examples are given in Figure 1), embody a speaker’s attitude toward the discussed object of discourse (e.g., Teßendorf, 2014; Bressem and Müller, 2017). The Throwing away gesture (Bressem and Müller, 2017), for instance, can be used to downplay an argument made by the speaker, the Brushing aside gesture (Teßendorf, 2014) may dismiss a point, and the Stretched index finger (Bressem and Müller, 2014) may highlight a crucial aspect.

Regarding the second aspect, works on social action and role inhabitance (e.g., Silverstein, 2004; Agha, 2006) have shown, that stances are often “indexical of enregistered identities” (Kiesling, 2022, p. 412). Thus, language, particularly speech styles, indexes stances that are simultaneously recognized as having the potential to shape identities (Johnstone, 2017; Kiesling, 2022). These styles are collections of linguistic forms that are associated with social norms, values and beliefs linked to specific ideologies that are familiar to members of a speech community. These linguistic forms circulate within that particular community. It will be shown that the gesture under investigation belongs to the repertoire of speakers engaged in public political communication. This leads to the final aspect explored in this paper, specifically the context of mediated political debates. It is demonstrated that the Slicing gesture is particularly employed by participants in televised political debates to enact stance and embody the rhetorical qualities of sharpness, decisiveness and commitment. To investigate this aspect, the paper focuses on a distinctive feature of political talk shows: the use of extended sequences of recurrent gestures. As will be shown, the Slicing gesture is frequently embedded within gesture sequences that include different variants of this gesture and/or other recurrent gestures. The focus on gesture sequences highlights another way of expressing stance. Stance can be conveyed through linguistic elements, such as the epistemic phrase *I think* as well as through the sequential occurrence of stance markers (Kärkkäinen, 2003, 2006). Lempert (2008, p. 572) referencing Jakobson’s (1960) poetic function, notes that “linearly co-occurring elements can be analyzed for their comparability, contrast and complementarity, likeness and unlikeness.” These occurring structures can “diagrammatically motivate pragmatic effects – including stance effects” by organizing the signs they consist of into comparable, sequentially ordered units (ibid.). The sequentially and comparability of co-occurring elements in gesture sequences offer a rich area for analyzing how gestures complement and contrast each other to convey nuanced stances together with speech.

The paper is structured as follows: It begins with an exploration of recurrent gestures in political communication, highlighting their role in presenting arguments and reinforcing rhetorical and social styles. The paper then delves into the specific dimension of taking stance with the recurrent Slicing gesture, discussing how it operates on single verbal elements within gesture sequences and develops meta-pragmatic meaning. Subsequently, the focus shifts to the kinesthetic experiences fundamental to the meaning of the Slicing gesture. The paper concludes with a discussion that synthesizes the findings and implications of the study.

## 2 Recurrent gestures in political communication

Recurrent gestures are characterized by their repeated occurrence with the same form and meaning across various communication contexts and among different speakers (Ladewig, 2014b, p. 31; Müller, 2017; Harrison and Ladewig, 2021; Ladewig, 2024). They are often derived from practical actions and are engaged in semantic and pragmatic meaning-making. In fact, the communicative potential of these kinds of gestures has always been a subject of study in the field of rhetoric (Ott, 1902; Mosher, 1916; Quintilian, 1969). Recurrent gestures, like any other gestures, are performed spontaneously, meaning their performance is not planned by a speaker. However, they differ from “singular gestures” (Müller, 2010, 2017), whose forms and meanings emerge while speaking. In the field of gesture studies, singular gestures are also called iconic gestures or metaphoric gestures (McNeill, 1992), “depictive gestures” (Streeck, 2009) or “representational gestures” (Kita, 2000). They have not undergone stabilization processes and are therefore not culturally shared as is the case with recurrent gestures and emblems.

Apart from getting insights into stabilization processes in communicative resources other than spoken or signed languages, recurrent gestures are an interesting research phenomenon because they are engaged in pragmatic meaning making. This aspect was first addressed from the point of view of rhetoric (Quintilian, 1969) and in the education of actors (Ott, 1902; Mosher, 1916). Following Kendon (2004, pp. 158–159) and other researchers, recurrent gestures can fulfil the following functions: (a) *modal*, i.e., framing how an utterance should be interpreted, (b) *performative*, i.e., enacting a speech act such as offering ideas or stopping someone, (c) *parsing*, i.e., punctuating the spoken discourse into logical components, and (d) *interactive and interpersonal functions* regulating turns at talk such as holding the floor or requesting a turn. These pragmatic functions make recurrent gestures a semiotic resource par excellence in the field of political communication, where the focus is on discussing arguments and sound reasoning. This hypothesis is corroborated by the study presented, which shows that 81% of gesture use consists of recurrent gestures in a five-hour data corpus of political talk shows (Table 1). In fact, recurrent gestures have already been described as a public event over more than 2000 years ago. As Müller points out ‘[g]estures have been considered a public phenomenon over 2000 years of European gesture study, and have been analyzed, stylized, and taught by scholars primarily for their impact on an audience’ (Müller, 1998, p. 30, translation S.L.). The description and training of gestures of speakers goes back to Cicero and Quintilian who regarded speech-accompanying gestures as worthy of cultivation (Müller, 1998; Kendon, 2004). Quintilian (1969) distinguishes between gestures which “naturally proceed from us simultaneously with our words” and “others which indicate things by means of mimicry” (XI, III.89). Almost all of the gestures Quintilian lists either mark speech acts, such as “demand, promise, summon, dismiss, threaten, supplicate […] question or deny” (XI, III.88), relate to parts of speech, or express emotions and attitudes, such as “indicating joy, sorrow, hesitation, confession, or penitence” (XI, III.88). The forms of the gestures he meticulously describes are reminiscent of the recurrent gestures identified in modern gesture studies, which have pragmatic functions and are integral to verbal utterances. Among the gestural forms described are the Palm up gesture (Kendon, 2004; Müller, 2004), the Ring gesture (Kendon, 2004; Neumann, 2004), the Fist (Bressem and Müller, 2014) or the Finger bunch (Kendon, 1995, 2004).

**Table 1:** Distribution of gesture types in the data.

Recurrent gestures	2,747
Hybrid recurrent gestures	89
New candidates for recurrent gestures	158
Recurrent gestures in total	2,994
Pointing gestures	237
Singular gesture	281
Unclear cases	104
Emblematic gestures	34
Beat gestures	8
Gestures in total	3,658

More recent studies of gestures in the field of political communication explore the role of speakers’ gestures and the attitudes they express in self-presentation, placing greater emphasis on this aspect over the purely interactive functions of gestures. Streeck’s (2008) study of the gestures used by Democrats in the 2004 U.S. election campaign ties in with Quintilian’s work. He documents a repertoire of recurrent gestures used by the members of the Democratic Party which, according to him, appeared to be reminiscent of a shared code or a “public gesture style” (Streeck, 2008, pp. 156, 178). These gestures primarily serve the function of visualizing the information structure of speech and thus help processing information. The gestures identified show “a surprising congruence between the type of gestures that Quintilian advocated” and “what appears to be an unspoken consensus about adequate gesticulation among the Democratic Party politicians” (Streeck, 2008, p. 178). One motivation for the emergence of such a way of gesturing is that the politicians are eager to keep a rhetorical style which may go along with the creation of persona in para-(social) interaction. Based on these observations Streeck argues, that the study of recurrent gestures in the field of political discourse may provide insights into the “theory of self-presentation (…) within the context of electoral politics” (Streeck, 2008, p. 183 referring to Goffman, 1956). This aspect is particularly relevant to the study presented in Section 3.2.2, where it is argued that the Slicing gesture develops the metapragmatic meaning of defining things with clarity and sharpness within extended gesture sequences. Consequently, the Slicing gesture can be considered a significant semiotic resource for presenting oneself with distinct rhetorical qualities.

Another compelling analysis of the use of recurrent gestures was conducted by Lempert (2011). He investigated Barack Obama’s use of the Ring gesture (Kendon, 2004; Neumann, 2004; Müller, 2014a) in his Senate race (2004), the primary debates (2007–2008) and the presidential debates against the Republican candidate, John McCain. Interestingly, Lempert noted a shift in the first presidential debate where Obama appeared to stage himself as a sharp speaker not only verbally but also gesturally. The Ring gesture (referred to by Lempert as precision grip) became a semiotic resource for Obama to not only make a sharp point but to present himself as “*being* sharp” (Lempert, 2011, p. 245, italics in the original). Thus, in a process of “reflexive reanalysis and conventionalization” (Lempert, 2011, p. 258), the Ring gesture has acquired a higher-order indexical value of being a sharp speaker which presupposes the lower-order focus of foregrounding a discursive object in his speech. In other words, the Ring has moved from focusing something *for* an addressee to making a sharp point *against* an addressee (ibid). “In semiotic parlance it may be termed a metapragmatic icon, to the extent that it reflexively (hence ‘meta-’) typifies communicative behavior as a social act (‘pragmatic’), and does so by means of felt resemblance (‘icon’-icity)” (Lempert, 2011, p. 258). Lempert notes that this process goes along with an indexical shift from working locally on parts of speech to pointing to a (candidate) brand that evolves interdiscursively. “The relevant units of analysis change as one moves through these orders as well, for the conditions under which ‘brand’ becomes recognizable, for instance, are quite different from those that motivate readings of speaker-persona” (Lempert, 2011, p. 262). In other words, the latter may become recognizable in the recurring uses of a gesture within one discourse where it not only visualizes recurring themes but also exhibits rhetoric qualities of the speaker. The former becomes possible in the relational field of political discourse (or of competition, as Lempert states, p. 259). Hence the “indexical icon of brand qualia” is an “interdiscursive precipitate” (Lempert, 2011, p. 245) that evolves over many political appearances.

To conclude, recurrent gestures can frame utterances, enact speech acts, punctuate discourse, and regulate interaction, making them indispensable in political debates for presenting arguments and reinforcing rhetorical and social styles of speaking and gesturing. The Slicing gesture, in particular, embodies decisiveness and clarity, highlighting its role as a semiotic resource in defining and emphasizing discourse positions. It will be explored in the following section.

## 3 Dimensions of taking stance with the slicing gesture

The Slicing gesture is the most common gesture in the data underlying this study. To be more precise in 5 h of mediated political discourse, i.e., German televised political talk shows, the gesture occurred 653 times over 27 speakers. However, this does not mean that all the speakers investigated are politicians. On the contrary, while four of the speakers are politicians, the rest are journalists and experts in political communication. What unites them in the data of televised talk shows is their engagement in publicly positioning themselves.

In the data, 3,658 gestures were documented, of which 2,994 are classified as recurrent gestures out of which 158 gestures are considered as candidates for the repertoire of recurrent gestures shown in Figure 1 but have not yet been included. Within these 5 h, we also recorded over 281 singular or depictive gestures, 237 pointing gestures, 34 emblematic gestures, and eight beat gestures. Additionally, 104 gestures were difficult to categorize and thus labeled as unclear (Table 1).

Figure 1 lists the distribution of different recurrent gestures over the data. For recurrent gestures already documented for German adults (Müller et al., 2013; Bressem and Müller, 2014), the most common gesture observed was the Palm up open hand (PUOH), occurring 619 times, followed by the Ring gesture (Kendon, 1995, 2004; Neumann, 2004) with 222 instances. The Cyclic gesture (Ladewig, 2014a) was noted 147 times, and the Holding away gesture appeared 122 times. The Stretched index finger was recorded 91 times, while the Fist gesture was identified 67 times. Sweeping away gestures (see also Kendon, 2004; Harrison, 2018, for English) occurred 64 times, Back and Forth gestures were observed 27 times, and Change gestures appeared 15 times. Brushing away gestures were recorded 14 times, Weighing up gestures were noted 18 times, Vague gestures were observed 8 times, Dropping of the hand gestures occurred seven times, and the Stretched index finger, moved horizontally, was recorded once.

For new recurrent gestures added to the repertoire, the Slicing gesture was the most common, observed 653 times. The Directing gesture (see Kendon, 2004, for English; Fricke, 2010, for German) appeared 292 times, while the Placing gesture (see also Tutton, 2011, for French and English) was noted 129 times. The Pressed thumb gesture was recorded 98 times, the Finger Bunch (see Kendon, 2004, for Italian speech) appeared 51 times, the Grip gesture (see Streeck, 2017, for English) was identified 44 times, and the Holding down gesture occurred 40 times.

Among the 89 hybrid recurrent gestures (Table 1), the following distribution was observed (Table 2): Directing combined with Palm up open hand occurred 46 times, the Holding away gesture combined with Sweeping away was noted 11 times, and the Bunch combined with Cyclic gesture was observed eight times. Additionally, the Slicing gesture combined with the Palm up open hand was documented eight times, the Slicing gesture combined with Directing appeared seven times, and the Pressed thumb combined with Cyclic gesture occurred three times. Furthermore, the Ring gesture combined with the Sweeping away gesture was observed 2 times, the Bunch combined with the Pointing gesture was noted once, the Slicing gesture combined with the Cyclic gesture was identified once, and the Slicing gesture combined with the Pointing gesture was recorded once.

**Table 2:** Distribution of hybrid recurrent gestures in the data.

Directing + Palm up open hand	46
Holding away + Sweeping away	11
Bunch + Cyclic gesture	8
Slicing gesture + Palm up open hand	8
Slicing gesture + Directing	7
Pressed thumb + Cyclic gesture	3
Ring gestures + Sweeping away	2
Bunch + Pointing gesture	1
Slicing gesture + Cyclic gesture	1
Slicing gesture + Pointing gesture	1

The gesture under investigation has been previously recognized in studies of adult French speakers (Calbris, 2003, 2011, calling it Cutting gesture, Rigid Hand or the both-handed Frame gesture) and English speakers (Streeck, 2008 calling it “the Slice”; Lempert, 2017; Harrison, 2018 calling it “chopping”). While Calbris, Streeck, and Lempert documented many instances of this gesture in the context of public and political communication, Harrison only documented it once in his data corpus of non-public conversations.^1^ Morris (1994) also documented one variant of this gesture, referring to it as the Hand Chop, which is used to cut through an argument. He describes the most conventionalized variant of this gesture where one “stiff hand chops down on the upturned palm of the other hand” (p. 103). This variant was documented nine times in the data presented. According to Morris, the derivational base of this gestural form lies in miming the action of cutting downwards with an axe or imitating a karate chop. He observes that this gesture is often employed in heated debates when someone wants to make a strong and clear point. Additionally, Morris notes a “pragmatic affinity” (Lempert, 2017) with the clenched fist slammed down, “but the chopping action reflects a mood of greater precision” (Morris, 1994, p. 103).

### 3.1 Exploring the slicing gesture: quantitative analysis of its forms and functions

The investigation of the Slicing gesture proceeded as follows. First, a data corpus was established and all gestures were identified and annotated using the annotation software ELAN (Wittenburg et al., 2006). The frequencies of all recurrent gestures, both previously documented (Müller et al., 2013; Bressem and Müller, 2014) and undocumented, were subsequently determined resulting in the repertoire shown in Figure 1. As the Slicing gesture emerged as the most frequently occurring gesture in the data corpus that had not been previously documented for German spoken language before, it was subjected to a more thorough analysis. This involved annotating the gesture with regard to its forms and functions in relation to speech, following the Linguistic Annotation System of gestures (LASG, Bressem et al., 2013). Based on that the core form and form variants of the gesture were determined. In the next step, the gesture’s functions were specified in relation to stance-taking categories, as the gesture was frequently embedded in contexts of positioning. The results of the analysis are as follows.

**Figure 2:**
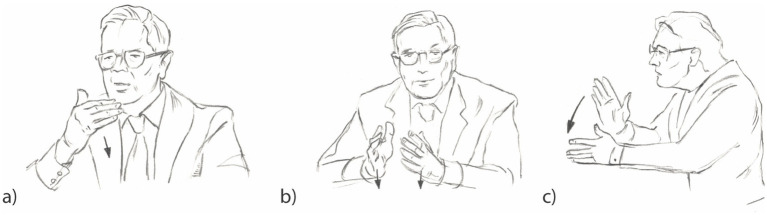
Variants of the Slicing gesture documented in the data of German political talk shows: **(a)** the Slicing gesture oriented towards the speaker’s body (PLTB), **(b)** the Slicing gesture oriented diagonally towards the speaker’s central gesture space (PLdiTC), and **(c)** the Slicing gesture aligned with the sagittal plane (PLTC).

The core form of the Slicing gesture is characterized by an open hand often with the palm facing toward the speaker’s body (117 cases, Figure 2A), facing diagonally the speaker’s central gesture space (114 cases, Figure 2B), or being aligned with the sagittal plane (338 cases, Figure 2C). Other less frequent variants include a two-handed Slicing gesture, with the palms facing the speaker’s central gesture space, as shown in Figure 2C, but directed to the left or right (54 occurrences). Orientations that appeared fewer than five times were categorized under “other orientations,” with a total of 30 occurrences.

Another form parameter contributing to the core form is the movement direction, which is downward in a straight or arced pattern. The gesture “evokes the act of cutting something into pieces or slices. The edge of the hand laterally cuts up sections of space “as Calbris (2003, p. 31) propose. The core meaning of Slicing and thus singling out an entity has emerged from the action of slicing in which the hand symbolizes a blade or is a manual blade. This aspect is illuminated in Section 4.

**Figure 3:**
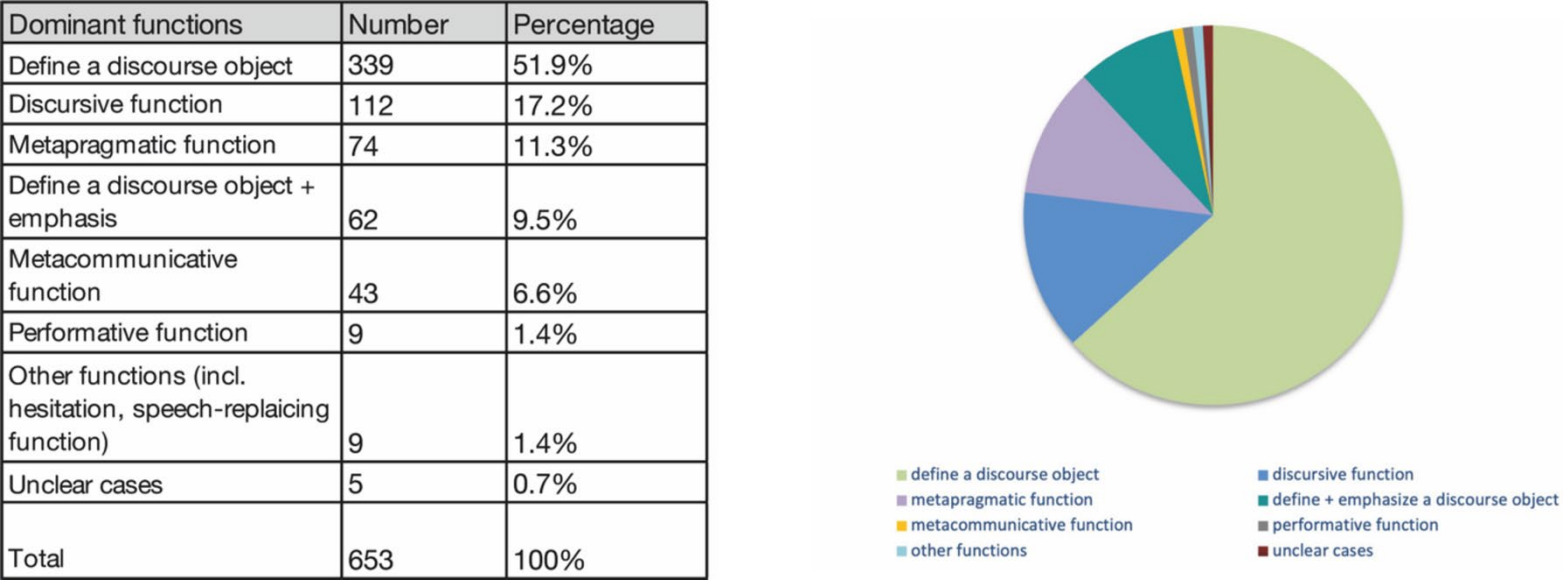
Most common functions of the Slicing gesture used in German televised political debates.

For the Slicing gesture used in televised political debates, the functions shown in Figure 3 were determined, with the most frequently observed being the definition of a discourse object, accounting for 339 occurrences (51.9% of the cases). Discursive objects can include ideas or arguments with which the speaker positions themselves in the discourse (see Section 3.2.1). The discursive function, involving the intensification of arguments through emphasis, was observed in 112 occurrences (17.2% of the cases). The metapragmatic function, where the gesture not only defines discursive objects but also embodies the speaker’s rhetorical act of clearly defining things for co-participants, appeared in 74 occurrences (11.3% of the cases) (see Section 3.2.2). A combined function of defining a discourse object and adding emphasis was present in 62 occurrences (9.5% of the cases). The metacommunicative (pragmatic modal) function, used to mark information structure or engage in speech activities such as enumeration, accounted for 43 occurrences (6.6% of the cases).

The performative function, where the gesture is used to perform speech acts, was documented in only 9 occurrences (1.4% of the cases). Other functions, including hesitation or substitution of speech, also accounted for 9 occurrences (1.4% of the cases). Some functions could not be clearly determined and were marked as unclear, constituting 5 occurrences (0.7% of all cases).

**Figure 4:**
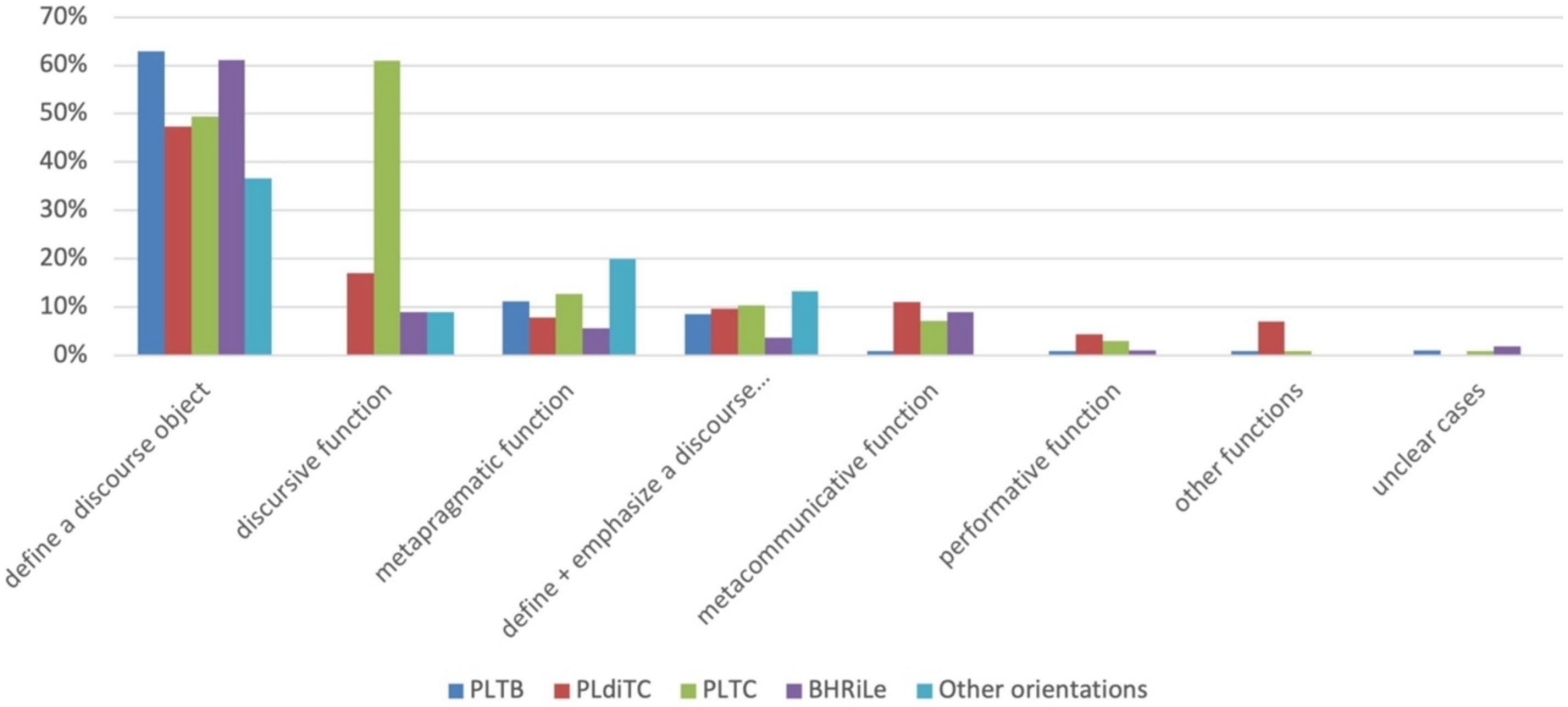
Distribution of functions by palm orientation. PLTB, palm lateral towards body (Figure 2A); PLdiTC, palm lateral diagonal towards center (Figure 2B); PLTC, palm lateral towards center (Figure 2C). The coding categories are based on Bressem (2013). BHRiLe, both hands facing each other, directing to the right or left with the palms in the sagittal plane.

Considering the orientation of the palm, identified as the feature defining form variants, the following distribution was observed (Figure 4). All orientations predominantly convey the meaning of defining a discourse object, with the highest percentages observed in the orientations palm lateral towards body (PLTB, Figure 2A), and palm lateral diagonal towards center (PLdiTC, Figure 2B). The second most common function across all orientations is the discursive function, followed by the metapragmatic function, particularly prevalent in the BHRiLe orientation (both hands facing each other, directing to the right or left with the palms in the sagittal plane). The combined function of defining a discourse object and emphasizing it is also notable in several orientations, while the metacommunicative and performative functions occur less frequently across all palm orientations.

One of the fundamental features of language is its capacity to concurrently depict subjects, objects, or events and convey a stance or perspective regarding these depictions (Andries et al., 2023). Similarly, gestures are multifunctional (e.g., Müller, 1998; McNeill, 2005; Kok, 2016), with the Slicing gesture exemplifying this versatility. This gesture indicates that speakers frequently engage in stance-taking beyond the functions already discussed. As Kiesling (2022, p. 417) notes, “stance is not simply a single utterance but rather emerges across multiple utterances.” This highlights how both language and gestures work together to express complex meanings and stances in communication. Consequently, a speaker may define a discourse object while evaluating it or positioning themselves regarding the topic under discussion. The speaker may also frame (or define) a question that invites stance-taking from other participants. Alternatively, the gesture might be used to highlight a crucial distinction within a conversation, effectively separating two perspectives. By emphasizing one perspective and diminishing the other, the speaker can position themselves in the discussion, drawing attention to the preferred viewpoint while sidelining the less favored one. For instance, when distinguishing between two competing theories, the Slicing gesture can underscore the speaker’s preference for one over the other by moving the hand toward the speaker’s body, subtly encouraging others to consider their stance. Thus, while addressing individual ideas verbally, the Slicing gesture simultaneously provide the marks these distinctions gesturally, providing a visual and physical representation of the separation and emphasis of different concepts.

**Figure 5:**
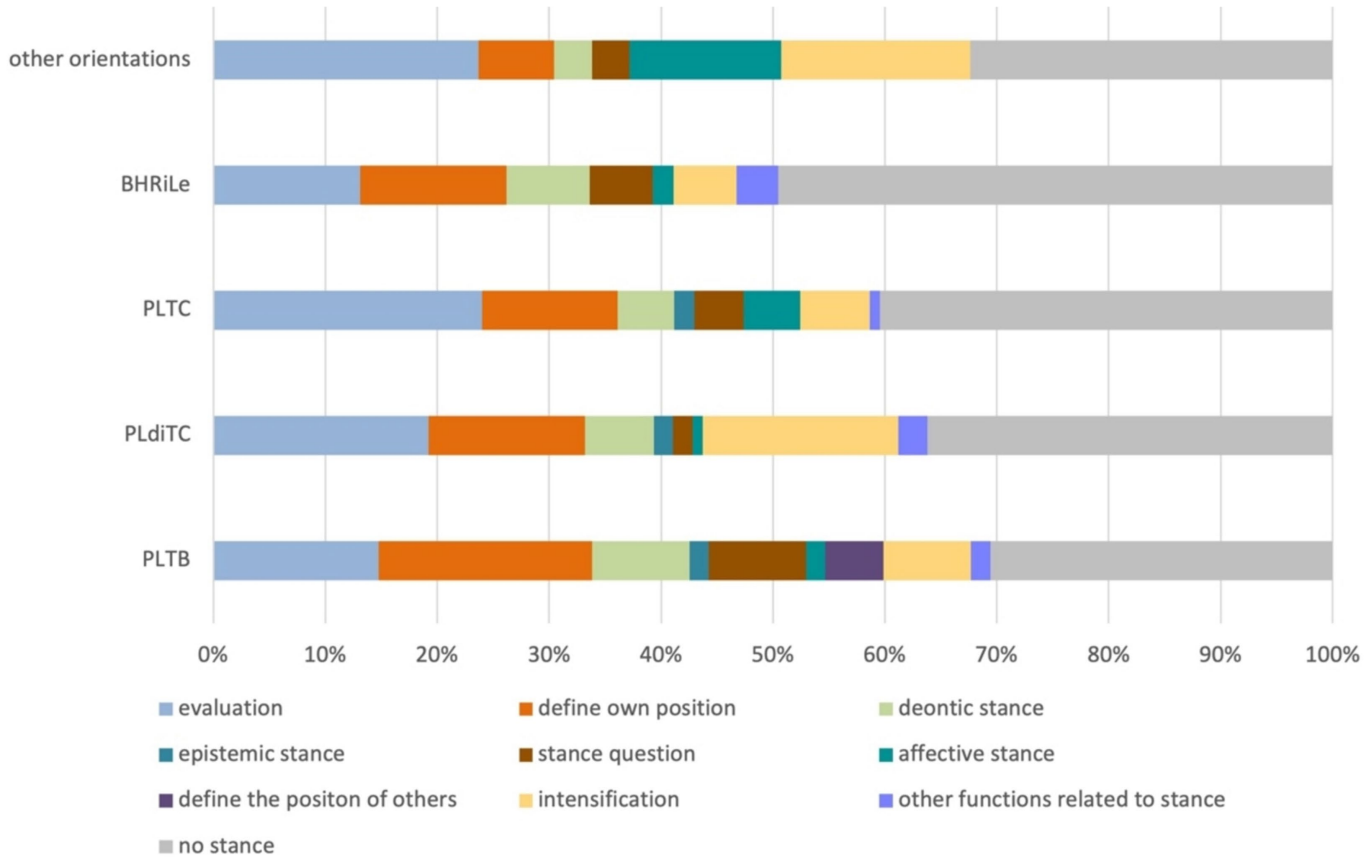
Functions of stance taking determined for the Slicing gesture and distribution over form variants. PLTB, palm lateral toward body (Figure 2A); PLdiTC, palm lateral diagonal towards center (Figure 2B); PLTC, palm lateral towards center (Figure 2C); BHRiLe, both hands facing each other, directing to the right or left.

Overall, the Slicing gesture is involved in stance-taking speech activity in 61% of the cases, equating to 401 out of 653 gesture occurrences. These cases can be further defined according to the different categories determined for stance taking (Figure 5).

Evaluation emerges as the most frequent function across all palm orientations, particularly in the palm lateral towards center orientation (PLTC, Figure 2C). This indicates that speakers commonly use the Slicing gesture to express evaluative stances, reflecting judgments or assessments about the discussed topic. The defining of one’s own position is also a prominent function, especially visible in orientations like palm lateral towards body (PLTB, Figure 2A) and palm lateral directed towards center (PLTC, Figure 2C). These orientations are frequently employed by speakers to assert personal stances that align with a group to which the speakers belong, such as a political party or professional group.

Deontic stance, which involves conveying obligations, permissions, and prohibitions, is consistently observed across orientations, though it is less frequent than evaluation. Affective stance, while less common, is still present across multiple orientations, indicating the gesture’s role in expressing emotions or attitudes.

Stance questions and intensification functions appear less frequently but are still noteworthy. Stance questions involve inquiring about another’s viewpoint, while intensification involves emphasizing particular points. Both are present across orientations in varying degrees.

Finally, other stance-related functions, such as distancing, are observed but are the least frequent across all palm orientations.

Overall, the Slicing gesture serves multiple functions in stance taking, with evaluation being the most prevalent across various orientations, followed by defining one’s own position and deontic stance. In what follows the focus of the paper will be shifted towards the qualitative analysis of the Slicing gesture, providing examples for some of the stance functions presented and highlighting different kinesic complexities and levels of gesture speech interaction.

### 3.2 Taking a stance with the slicing gesture: a qualitative analysis

Taking a stance through the Slicing gesture can manifest in various ways, depending on how it interacts with speech. In some cases, the gesture’s meaning is closely tied to specific verbal elements, emphasizing particular words or phrases and reinforcing the speaker’s point. In other instances, the Slicing gesture takes on a broader metapragmatic role, especially when used in extended gesture sequences. Here, it goes beyond individual utterances, embodying an overarching communicative strategy that helps define and clarify the speaker’s stance throughout the discourse. This section explores these different dimensions of stance-taking through qualitative analysis, revealing the versatility and impact of the Slicing gesture.

Building on the diverse ways the Slicing gesture can convey meaning, what stands out is its frequent embedding in gesture sequences. Specifically, it often appears (a) in sequences of varying recurrent gestures or (b) in sequences involving different variants of the Slicing gesture. This pattern seems to be unique to the mediated contexts observed in this study, as such extended gesture sequences are rarely found in private settings.^2^ Despite their prevalence in public discourse, gesture sequences remain an underexplored area in gesture studies. Exceptions include studies on recurrent gestures marking the topic comment structure of an utterance. Gesture sequences of this kind often show an “open-to” structure such as the a combination of a Ring gesture combined with an open Pistol hand (Seyfeddinipur, 2004) or the finger bunch or *grappolo* combined with a Palm up open hand (Kendon, 2004). In both cases, the sequences start with a specific handshape and movement, the Ring or the Finger bunch, marking the topic, and are followed by an opening of the hands as a comment on the topic given.

Harrison (2018) gives a more comprehensive account of sequences of recurrent gestures associated with negations. He defines these sequences as patterns of two gesture phrases within one gesture unit where the transition of the gestures involves the rotation of the wrist. The observed “sequences occur over utterances that ensue as part of a single rhetorical move, speech act, or argument, usually within the confines of a single turn at talk” (Harrison, 2018, p. 105). Moreover, Harrison observed that the rotation of the wrist reflects shifts at the level of discourse. Examples include the “Palm Up to Horizontal Palm Gesture Sequence” (Harrison, 2018, p. 108) which (a) embodies a topic comment structure, (b) is part of a conditional statement in which the gestures accompany the information structure of such statements, or (b) relates to a verbal context that precedes the gestures sequence while the gestures occur in absence of speech.

Gesture sequences are not only composed of different recurrent gestures but can also be formed by the repeated use of recurrent gestures. Some examples are given by Calbris (2003) who suggests that the repeated use of the Cutting gesture in French marks “the successive consideration of elements as they are cut out “(p. 43) or the singling out of objects (p. 33). Müller (2004) noted that the repeated downward movement of the Palm up open hand can be used to list a series of arguments. Similarly, Bressem (2021) documented the repetitive use of recurrent gestures that have a prosodic function.

The behavior of the Slicing gesture in gesture sequences will be explored in the following sections, with a focus on the process of multimodal meaning-making. Particular attention will be given to the various dimensions of stance-taking involving the Slicing gesture in sequences of its different variants, of different recurrent gestures, and different gesture types. Additionally, the Slicing gesture’s conveyance of meta-pragmatic meaning in extended gesture sequences will be examined.

#### 3.2.1 Operating on single verbal elements within gesture sequence of recurrent gestures

This section mainly focuses on examples in which the Slicing gesture operates on single verbal constructions of an utterance. This means that the gesture is closely linked with specific words or phrases within a sentence, thereby reinforcing the speaker’s point. The chapter is organized based on the different gesture sequences observed in the data, including sequences of various variants of the Slicing gesture, sequences of different recurrent gestures, and sequences of different gesture types in which the Slicing gesture is embedded.

##### 3.2.1.1 Sequences of different variants of the slicing gesture

**Figure 6:**
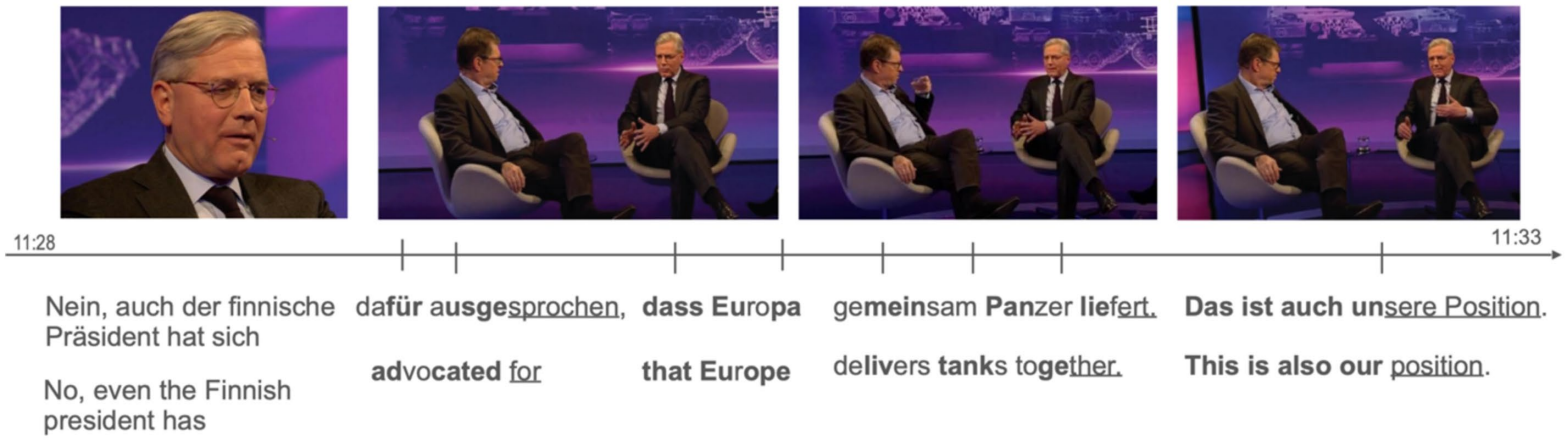
Example of a gesture sequence embedding different variants of the Slicing gesture. Stroke phases are marked bold, post-stroke holds are underlined, and preparation phases show no formatting. The images are sourced from an interview on the German TV show “Maischberger,” aired on January 18, 2023. Link to the interview: https://www.youtube.com/watch?v=8BdJY4RGfuk.

In the first example, the Slicing gesture is embedded in a sequence of its different variants. This example is taken from the talk show “Maischberger,” where two politicians are discussing their views on the war in Ukraine. The sequence shown in Figure 6 is a response to the host’s question, *Ist das so?* (‘Is that the case?’). This question addresses the accusation made by the speaker sitting on the left in Figure 6, suggesting that statements from political allies are consistently interpreted as supporting their own political stance.

By saying *Nein* (‘No’) the speaker shown in the first image in Figure 6 clearly rejects this accusation. He continues by stating that the Finish president has advocated for a certain military strategy in support of Ukraine against Russia. With these verbal utterances the speaker clearly aligns with a significant political viewpoint, reflecting both a shared understanding and an endorsement of collective action. By invoking the Finnish president’s advocacy, the speaker bolsters their argument by associating it with a high-profile political figure, thereby not merely stating a fact but emphasizing the necessity and appropriateness of the proposed action of delivering tanks. The inclusion of a direct statement of agreement, *Das ist auch unsere Position* (‘This is also our position’), further solidifies the stance by transitioning from merely reporting an opinion to personally endorsing it. This transition is crucial as it positions the speaker within a specific political and ethical framework that advocates for proactive measures in defense matters. The use of the adverb *auch* (‘also’) and the possessive pronoun *unsere* (‘our’) implies a collective agreement or policy stance, indicating the speaker’s affiliation with the political party to which he belongs, a party that shares this perspective.

The speaker’s explanations are co-expressed with a series of Slicing gestures. The first part of his multimodal response features eight two-handed Slicing gestures, with the palms oriented laterally and diagonally within the speaker’s gesture space. As shown in the transcript in Figure 6, these manual movements align with specific parts of the speaker’s utterance, defining the Finnish president’s position and underscoring relevant sections of his statement. The downward movements synchronize with the prosodic prominence in the verbal utterance, specifically with the stress on certain syllables.

The sequence of unstressed and stressed syllables manifests in the upward movement of the hands during the preparational phase and the downward movement during the stroke phase, creating a perceptual rhythm of uniform but emphatic accentuation. This alignment between speech and gesture not only intensifies the semantic content also conveys affective meaning, forming a coherent multimodal emphasis that unfolds temporally.

This rhythmic pattern is interrupted when the speaker explicitly references the position taken by his own party in the debate on delivering tanks to Ukraine. Shifting from using both hands to one hand and changing the orientation of the palm to face his upper body, the speaker states, *Das ist auch unsere Position* (‘This is also our position’). This shift in focus is accompanied by three parameters: handedness, palm orientation, and movement pattern, as the speaker executes an arc-like movement directed toward his body. Additionally, the speaker does not repeat the gestural movement but executes a single gestural stroke, as shown in the final image of Figure 6. These kinesic features make the gesture stand out from the previously created perceptual gestalt, embodying the attentional and discursive shift from the Finnish president to the position of the speaker’s party including the speaker himself.

This observation aligns with Harrison’s findings on wrist rotation reflecting shifts at the discourse level, such as the “Palm Up to Horizontal Palm Gesture Sequence” (Harrison, 2018, pp. 108–114). The observations presented here build on this research, suggesting that a shift in hand orientation indicates a shift in the speaker’s focus within the discourse, particularly towards supporting their own position. The latter complements Calbris (2003, 2011) thorough analysis of the Cutting gesture in French, suggesting that movements along the sagittal axis represent ideas or entities on an interpersonal level, with the speaker committing to one of these ideas. When the gesture moves further away from the speaker’s body, it signifies the ideas of another party who may be present.^3^ Conversely, if the gesture is close to the speaker’s body, it represents ideas or views with which the speaker agrees. This personal character is established by an.

experiential link (…) between the axis of walking and personal progression or action: it is on this axis that the actor responsible for the action (…), or who is impeded in carrying out his action (…) is situated. This link is itself deduced and confirmed by the confrontation with other examples that include the gestures (…) described here and other gestures characterized by a movement forwards (Calbris, 2003, p. 40).

In the analysis presented, 32 instances of the Slicing gesture directed toward the speaker’s body were observed in conjunction with the use of the first-person pronoun. These gestures occurred when speakers were formulating their own position or the position of a group to which they belong, such as a political party or professional group.

##### 3.2.1.2 Sequences of different recurrent gestures

**Figure 7:**
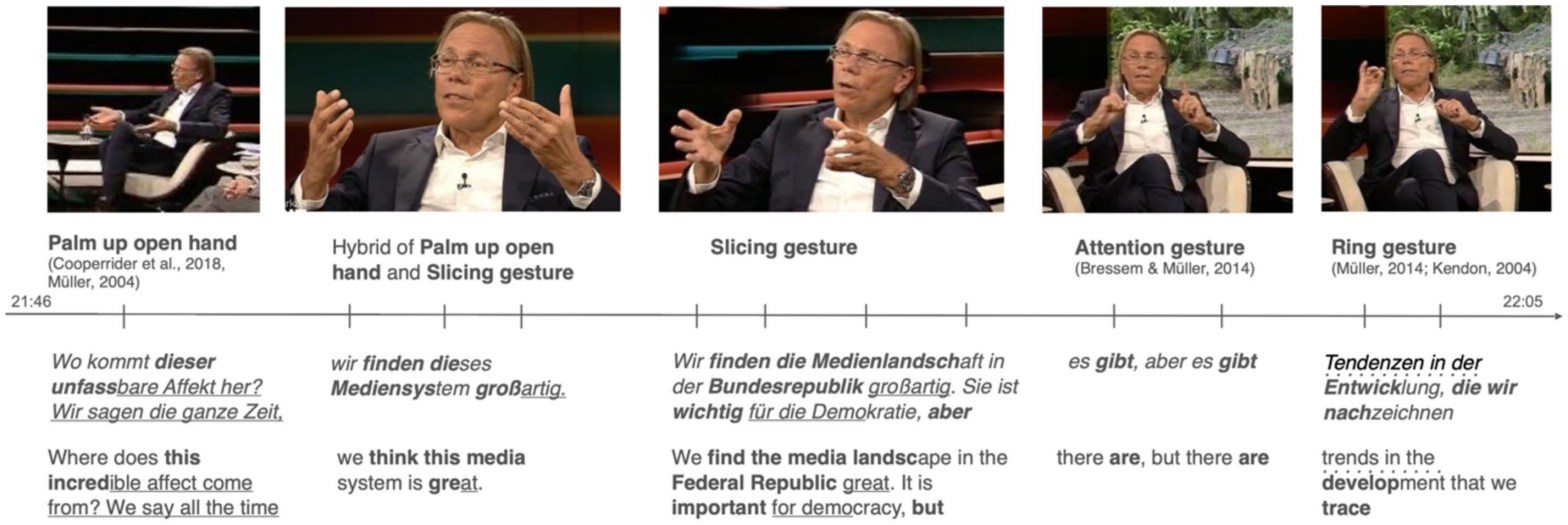
Example of a gesture sequence embedding different recurrent gestures. Stroke phases are marked bold, post-stroke holds are underlined, pre-stroke holds are marked with a dotted underline, and preparation phases show no formatting. The images are sourced from the German TV show “Markus Lanz,” aired on September 29, 2022. Link to the show: https://www.zdf.de/gesellschaft/markus-lanz/markus-lanz-vom-29-september-2022-100.html.

In the example, illustrated in Figure 7, the speaker discusses the public opinion regarding the book he has co-authored, elaborating a critical stance towards the media in Germany. The verbal utterances provide a nuanced example of stance taking, characterized by both affective and evaluative dimensions. These utterances are co-expressed with a sequence of recurrent gestures, within which the Slicing gesture is embedded.

The gesture sequence in question occurs during a discussion about the thesis of a book co-authored by the speaker, as shown in Figure 7. The book critically examines media coverage of the so-called refugee crisis and the war of aggression against Ukraine in Germany. It starts with a two-handed Palm up gesture (Kendon, 2004; Müller, 2004) which is performed with a large movement (first image in Figure 7). It is co-expressed with the rhetorical question *Wo kommt dieser unfassbare Affekt her?* (‘Where does this unbelievable affect come from?’), expressing surprise and critique about the intensity of the emotion towards the media reflection published in his book. The speaker’s opening question sets the stage for his stance by expressing bewilderment and inviting reflection on the source of a profound emotional response. The use of the adjective *unfassbar* (‘unbelievable’) intensifies the affective stance, indicating that the speaker finds the level of emotion not only noteworthy but also excessive and potentially problematic. By questioning the origin of this affect, the speaker implicitly positions himself as someone who finds this emotional response unusual and worthy of critical examination.

The both-handed Palm up gesture accompanying the rhetorical question is an instance of the “palm-up epistemic” variant (Cooperrider et al., 2018), described by many authors to convey a consistent range of epistemic meanings (e.g., Chu et al., 2014; Debras, 2017; Mittelberg, 2017; Marrese et al., 2021). This gesture has been associated with epistemic stances related to the absence of knowledge and the subtypes derived from this origin, including uncertainty, obviousness, and interrogatives. The latter is the case observed here, where the gesture accompanies a rhetorical question (Kendon, 2004). Furthermore, the large movement with which the stroke is performed, characterized by the opening of the hands and a downward motion, embodies the affective meaning of bewilderment and emphasizes the speaker’s incredulity.

In the following utterance the speaker turns to the position advocated in his co-authored book: *Wir sagen die ganze Zeit, wir finden dieses Mediensystem großartig. Wir finden die Medienlandschaft in der Bundesrepublik großartig* (‘We say all the time that we think this media system is great. We think the media landscape in Germany is great’, Figure 7). Here, the speaker adopts an evaluative stance by repeatedly asserting the excellence of the media system and landscape in Germany (*großartig*, ‘great’). The repetition of *wir finden* (‘we find’) emphasizes a joint endorsement by the speaker and his co-author, who is also present in the talk show, suggesting that their view is shared within their collaborative perspective. This collective stance not only strengthens the evaluation but also serves to justify their book against critique, which suggests that the authors criticize the work of the media. By presenting their positive viewpoint as a consistently held and well-established position, the speaker counters the critique leveled against their book. What is more by stating that the media is crucial for democratic functioning, the speaker elevates the significance of their previous positive evaluation thus positioning the speaker firmly in support of the current media landscape.

Notably, the shift in the discursive object from the public’s perspective to the authors’ stance is mirrored by a corresponding shift in the gestures. The first utterance, *Wir sagen die ganze Zeit, wir finden dieses Mediensystem großartig. Sie ist wichtig für die Demokratie* (‘We say all the time that we think this media system is great. It is important for democracy’), is accompanied by a hybrid form of the recurrent Palm up open hand gesture and the Slicing gesture, as illustrated in the second image in Figure 7 (see also Table 2). This “hybrid gesture” (Morris, 2002, p. 58) merges the core meanings of both gestures: defining the authors’ position while presenting it on a more or less open hand.

The subsequent utterance, *Wir finden die Medienlandschaft in der Bundesrepublik großartig. Sie ist wichtig für die Demokratie* (‘We think the media landscape in Germany is great. It is important for democracy’), is accompanied by multiple strokes of the Slicing gesture, shown in the third image of Figure 7. These gestures not only establish the media system as a discursive object but also emphasize the authors’ positive stance toward it.

The following limitation of this positive evaluation is accompanied by the Stretched index finger, a gesture described as expressing attention (Müller et al., 2013; Bressem and Müller, 2014). This gesture marks the contrasting stance established in speech, signaling a shift from a general praise to a more nuanced position. By acknowledging ‘tendencies in development’ (*Tendenzen in der Entwicklung*) that require attention, the speaker adopts a critical stance. This critical stance does not negate the earlier positive evaluations but adds complexity to the speaker’s position, indicating awareness of and concern for ongoing changes that might threaten the media’s positive role. This more nuanced and critical position is embodied by the Ring gesture (Müller, 2014b) the speaker uses twice, which conveys the idea of making a precise point.

The speaker’s stance in these utterances is multifaceted, combining strong positive evaluations with a cautious critical perspective. The single gestures used in this gestural sequence are highly coordinated and aligned with ideas expressed in speech. The two-handed Palm up gesture expresses bewilderment and critique, while the hybrid Palm up open hand and Slicing gesture emphasizes positive evaluations. The transition to a critical stance is marked by the Attention gesture (Stretched index finger), and the Ring gesture underscores specific concerns, effectively mirroring the progression of the speaker’s verbal message. This integrated use of gestures and speech demonstrates the nuanced expression of complex stances in political discourse.

##### 3.2.1.3 Sequences of different gesture types

The following example illustrates the embedding of the Slicing gesture within a sequence of different gesture types including singular gestures (also known as depictive gestures or iconic gestures). In the example illustrated in Figure 7, the speaker juxtaposes different interpretations of the ban on violence in international law, arguing that the West bends the law to fit its own goals when necessary. As he begins to elaborate on his argument, he uses the Slicing gesture, which is embedded in a sequence of three gestures. Notably, each idea in his utterance is accompanied by a corresponding gesture.

**Figure 8:**
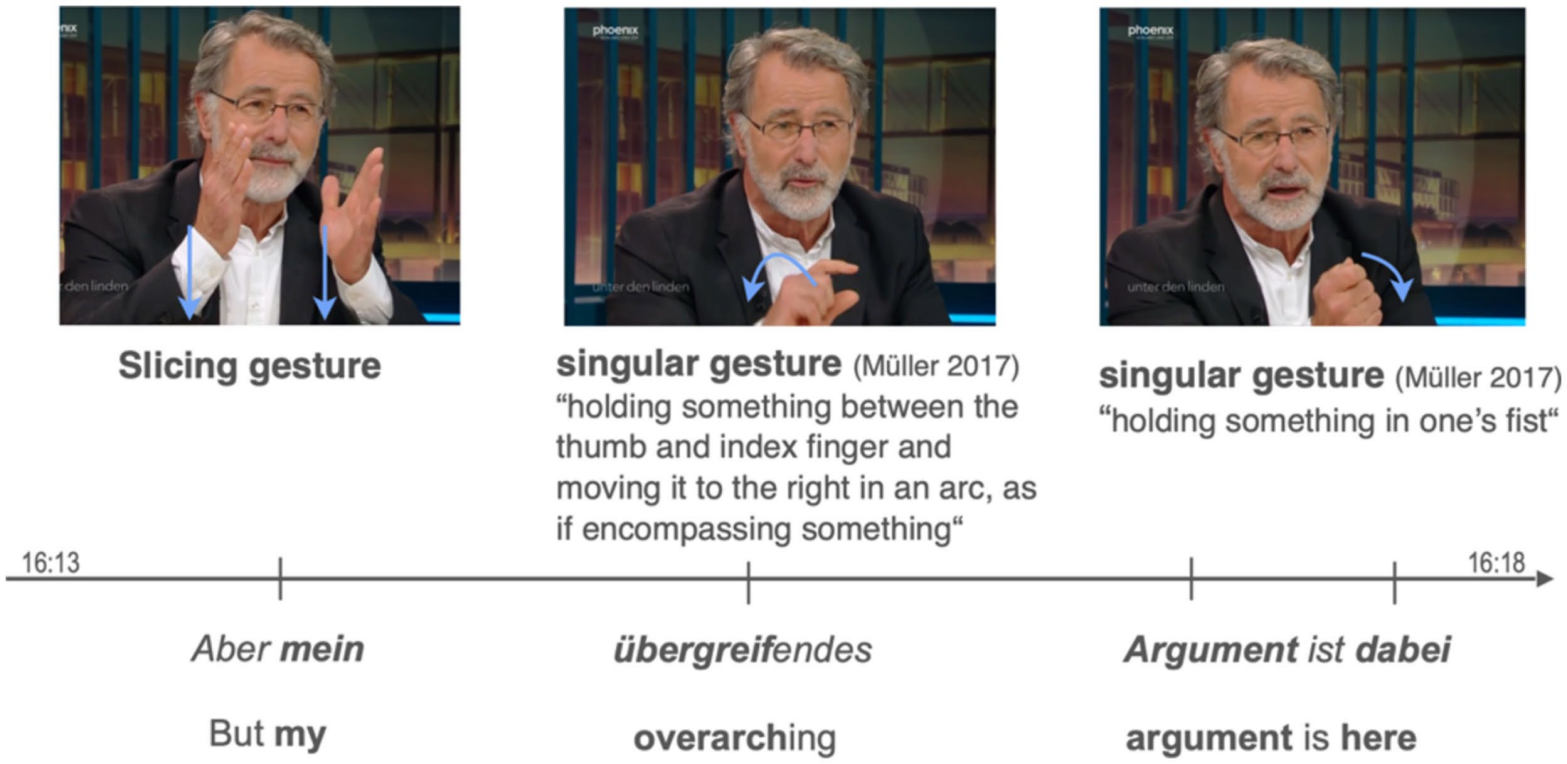
Example of a gesture sequence embedding different gesture types including the Slicing gesture. Stroke phases are marked bold, preparation phases show no formatting. The images are sourced from the German TV show “Unter den Linden,” aired on September 19, 2022. Link to the show: https://www.ardmediathek.de/video/unter-den-linden/wendepunkt-erodiert-putins-macht-chance-auf-frieden/phoenix/Y3JpZDovL3Bob2VuaXguZGUvMjkzMjU0OA.

The beginning of his utterance *Aber mein übergreifendes Argument dabei* ist (‘But my overarching argument here is’) is accompanied by the Slicing gesture. While producing the possessive pronoun *mein* (‘my’) the two-handed version of the Slicing gesture is performed. The hands are moved down while being placed in the central gesture space. The gesture essentially cuts out and isolates an idea (in this case, an argument that is verbally referenced later) and defines it. The gesture is followed by a manual movement showing a pincer-like configuration, where both the thumb and the index finger are spread apart and slightly bent (second image in Figure 8). The gesture is moved in an arc to the right of the speaker while he says *übergreifend* (‘overarching’; literally: ‘over-grasping’). The gesture basically embodies both morphemes of the adjective: *über* (‘over’) is depicted by the arced movement to the right as if the gesture spans several entities, *greifend* (‘grasping’) is embodied by the pincer-like configuration of the hand. The gesture involves a fist being repeatedly moved downward while the speaker says *Argument ist dabei* (‘argument here is’, see bold characters in the third image in in Figure 8 for the position of the strokes). This gesture depicts the holding of an object while emphasizing its strength as well as the speaker’s involvement in the discussion with the downward movement.

In this example, the Slicing gesture brings the speaker’s view on the handling of the international law into the focus of attention. By being simultaneously expressed with the possessive pronoun *mein* (‘my’), it essentially isolates (or cuts out) his argument from others and clarifies its definition. The subsequent gesture alludes to this argument being isolated and further elaborates on it both verbally and gesturally. The third gesture physically grasps the isolated argument in the hand, emphasizing it with a repetitive back-and-forth movement.

This example differs not only in the structural components, i.e., the gesture types, but also in its temporal dynamic. This sequence is characterized by a quick succession of gestures leading to the fact that almost each word is accompanied by a new gesture.

#### 3.2.2 Developing meta-pragmatic meaning in gesture sequences of slicing gestures

**Figure 9:**
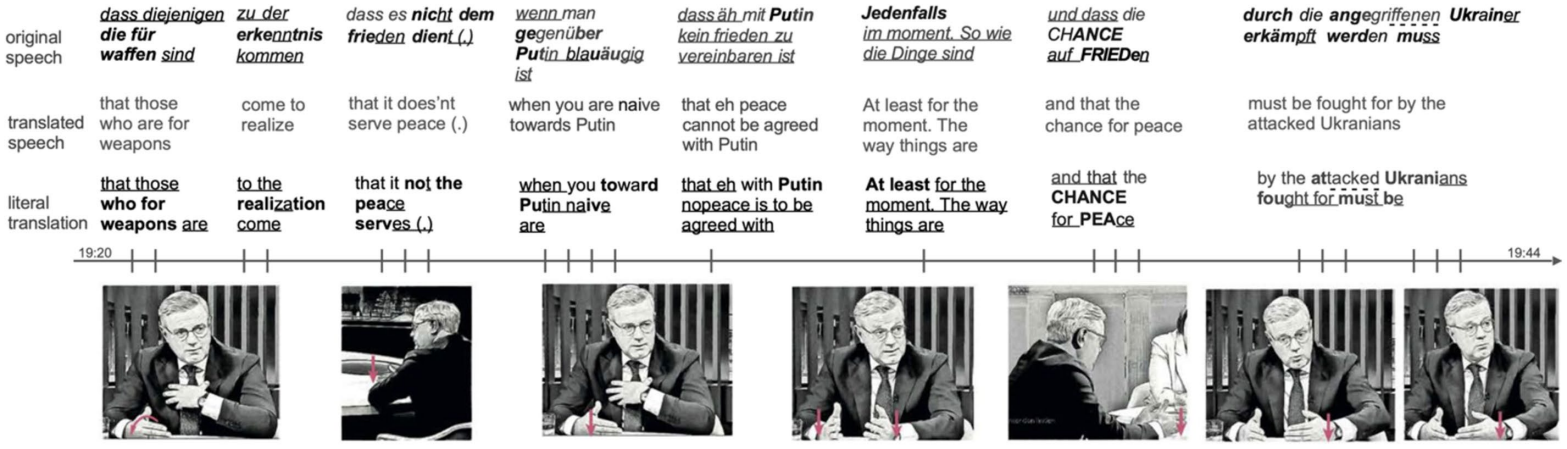
Example of an extended Slicing gesture sequence with metapragmatic meaning. Stroke phases are marked bold, post-stroke holds are underlined, pre-stroke holds are marked with a dotted underline, and preparation phases show no formatting. The example is sourced from the German TV show “Unter den Linden,” aired on September 19, 2022. https://www.ardmediathek.de/video/unter-den-linden/wendepunkt-erodiert-putins-macht-chance-auf-frieden/phoenix/Y3JpZDovL3Bob2VuaXguZGUvMjkzMjU0OA.

The examples presented in the previous sections show a tight interplay of gesture and speech in the meaning-making process. This means that the gestures discussed acted on individual parts of speech and form multimodal units with them. However, we also observed cases, in which the Slicing gesture became more detached from speech and thus appeared to have developed a second-order meaning. This phenomenon was particularly evident in cases of extended gesture sequences in which the gestural strokes exceeded five repetitions. An example is given in Figure 9.

The whole multimodal explanation lasts 24 s. Its temporal unfolding is visualized by the arrow in Figure 9. The single gestural strokes are placed on the arrow by means of vertical lines. Their accompanying speech units are set above it. While answering the question addressed by the host, the speaker uses 22 Slicing gestures strokes. In the majority of cases (16 gestures), the speaker uses a single hand with the palm oriented diagonally toward the center (PLdiTC, see also Figure 2B). In contrast, six gestures feature the palm facing the speaker’s body (PLTB, see also Figure 2A). In one case, the two-handed version is used which appears to be the only gesture in this sequence which aligns thematically with speech. To be more precise, in this instance, the Slicing gesture defines a moment in time when the speaker says *jedenfalls im moment* (‘at least for the moment’). The hands are moved down co-expressively with the adverb *jedenfalls* (‘at least’) and held while saying *im moment* (‘at the moment’). The other strokes of the Slicing gesture are aligned temporally with speech, but they do not operate on single discursive objects verbalized. This means they do not primarily define a moment in time or a discourse object. These versions of the Slicing gesture are employed by the speaker to consistently integrate his multimodal utterances thematically [catchment in McNeill’s (1992)], signaling their cohesive involvement in a single communicative activity. What’s more, they also visually convey the speaker’s intention to clearly define one of the views discussed in the talk show. The speaker’s alignment with this view is evident not only through his verbal expression but also through numerous Slicing gestures directed towards his body, reflecting an interpersonal level of argumentation.

The Slicing gestures in this example demonstrate a higher level of independence from individual speech components, operating on a meta-level. Rather than anchoring themselves to specific propositions or verbalized elements of the speaker’s discourse, the frequent use of this gesture creates a dynamic characterized by a broader rhetorical stance—one of clarity and sharpness, effectively ‘defining things clearly.’ As an embodied discursive strategy that characterizes communicative behavior as a social act (Lempert, 2011, p. 258), this metapragmatic meaning becomes particularly evident in sequences of the Slicing gesture. Thus, the analysis shifts from isolated speech or gesture elements to more extended, cohesive patterns of gesture use. These patterns, or longer sequences, signal a higher level of communicative intent, positioning the Slicing gesture as a powerful tool for shaping meaning and interaction beyond the immediate verbal content. This aligns with Lempert’s (2011) argument, highlighting the importance of considering larger units of analysis in understanding communicative behavior.

In essence, the Slicing gesture frequently occurs in sequences featuring various recurrent gestures or different variants of the Slicing gesture. In the former scenarios, this gesture is closely connected to the discursive objects verbalized in the utterances. When employed in extended sequences of Slicing gestures, it embodies the metapragmatic meaning of defining things with clarity and sharpness.

## 4 Kinesthetic experiences as fundamental to the meaning of the slicing gesture

The quantitative analysis of the Slicing gesture reveals its prevalent use in defining discourse objects and conveying metapragmatic meanings of sharpness and decisiveness. These functions are frequently integrated into stance-taking activities, including evaluative, deontic, and affective stances. The gesture’s suitability for these communicative purposes stems from its physical characteristics, which embody a “categorical or decisive character” (Calbris, 2011, p. 116). Specifically, the downward movement, the flat hand, which exposes edge of the hand, work together to symbolize a slicing action. This movement effectively demarcates concepts or delineates positions, thereby reinforcing the speaker’s decisiveness and clarity in their stance. It emphasizes a sense of finality or closure, which aligns with the decisive and categorical nature of the Slicing gesture.

The categorical aspect (…) stems from the perceptual schema of cutting, of separating: ‘that is it; that is not it’. By analogy with the cutting edge of a blade or an axe, lowering the edge of the hand relates back to cutting, physically and psychologically ‘slicing’, i.e., to deciding, to stopping something, to being categorical. By extension, the gesture evokes the categorical character of an assertion, a fact or a principle (Calbris, 2003). Thus, regardless of the configuration, the lowering of the edges of rigid, vertically held hands represents the action of slicing, separating, stopping and, hence, the act of deciding (Calbris, 2011, p. 121).

The act of deciding, Calbris refers to, can literally be seen as embodied by this gesture given the etymology of the word “decisive” whose stem is derived from the Latin word *decidere* which means “to cut off” (in the sense of decide)^4^. What is more, the Slicing gesture’s capacity in communicating sharpness and decisiveness is intimately linked with the quality of movement and thus with the sensorimotor experience of this gesture. Accordingly, signification resides in the gesture itself and the physical sensation of performing it. The speaker experiences these proprioceptive qualities firsthand, reinforcing their sense of actively defining and categorizing concepts. This embodied experience allows them to internalize the sensation of sharpness, decisiveness and authority. Consequently, the gesture not only communicates these aspects to the interlocutor but also reinforces the speaker’s own self-perception and stance, contributing to a second-order meaning of being sharp and decisive in their communication.

The proprioceptive dimension referred to here, also plays a crucial role in understanding the meaning of this gesture. As Calbris (2003) emphasized regarding the Cutting gesture in French, the interlocutors empathically perceive the proprioceptive qualities of the gesture, such as muscular tension and control. The abrupt nature of an individual’s Slicing gestures induces similar sensations of rigidity and sudden motion in the interlocutor’s body. The characteristics of power, control, and sharpness signaling determination, boundaries, or emphasis within the context of discourse are not only felt by the speaker but also by the interlocutor, making understanding a gesture a not only a visual but physical experience.^5^

This dimension of meaning-making has often been overlooked in gesture studies that prioritize gestures’ visuo-spatial imagery. In this traditional view, gestures represent actions or objects through their iconicity, with production and understanding depending primarily on visual properties. However, the idea that gestures convey meaning through the manual actions they are based on (e.g., Kendon, 2004; Müller, 2004; Streeck, 2009) already challenges the dominance of imagery in the meaning-making process. Recent research increasingly highlights the proprioceptive dimension of gestures and other body movements (e.g., Müller and Ladewig, 2013; Streeck, 2017; Müller and Kappelhoff, 2018; Ladewig and Hotze, 2021), emphasizing its central role in the meaning-making process of gestures. Accordingly, the meaning of being sharp, decisive and clear not only relies on visual qualities of the gesture but is particularly related to the “embodied-affective aspects” (Gallagher, 2017, p. 152) of the configuration and movement of the hand. “The uniqueness of the dynamics is first and foremost a kinesthetic uniqueness, not a visual uniqueness” (Sheets-Johnstone, 2003, p. 74). Therefore, the body is never absent or transparent but contributes to the speaker’s and interlocutors experience of a situation (see Colombetti, 2014). Movements are felt and can be controlled, and they are understood through the body (ibid., see Kappelhoff and Müller, 2011; Horst et al., 2014; Müller and Kappelhoff, 2018; Ladewig and Hotze, 2021, for phenomenological approaches to gestures that have evolved in recent years). The kinesthetic experiences evoked by the gestural form are fundamental to its meaning and help stabilize its interpretation (see Sheets-Johnstone, 2003). The gesture’s proprioceptive qualities—such as power, control, tension, and sharpness—play a crucial role in its communicative function. The hand, acting as a manual blade, performs a cutting motion that isolates and highlights specific concepts and viewpoints. This act of delineation makes the gesture an exceptionally potent semiotic resource for expressing a stance. By embodying the physical sensations associated with decisiveness, sharpness and clarity, the gesture effectively conveys the speaker’s commitment to a discussion, making their position clear to the audience.

## 5 Conclusion

This paper investigated the use of the Slicing gesture in mediated political communication, particularly focusing on German televised political talk shows, with a primary emphasis on stance-taking. The Slicing gesture is identified as a recurrent gesture frequently used to define discourse objects and convey sharpness and decisiveness. These meanings are embodied by the gesture’s physical characteristics, such as its downward movement and flat hand configuration, which symbolize a slicing action that demarcates concepts and positions, reinforcing the speaker’s decisiveness and clarity.

Quantitative analyses reveal that the Slicing gesture is embedded in the speech activity of stance-taking, encompassing evaluative, deontic, and affective stances. In expressing these versatile meanings of stance, the Slicing gesture is often integrated into gesture sequences, which can include different variants of the Slicing gesture, various recurrent gestures, or different gesture types. These sequences are particularly prevalent in mediated contexts, such as political talk shows, where longer gesture sequences are more common than in private settings. Also extended sequences of variants of the Slicing gesture are common in televised political communication. In these cases, the gesture appears to develop momentum, no longer operating on the level of single parts of speech but adding a metapragmatic dimension to the discourse. The metapragmatic meaning ‘defining things with clarity and sharpness’ is diagrammatically motivated by the juxtaposed Slicing gestures, creating a perceptual gestalt that reinforces the speaker’s engagement and stance in the debate demonstrating that taking stance is “an essentially *interactive activity*” (Kärkkäinen, 2003, p. 183, emphasis in the original). What is more, the speaker present themselves in this way as someone who clearly names things and positions themselves in the discussion by differentiating from their fellow discussants, thereby taking an interpersonal stance.

The study argued that the meaning-making process of the Slicing gesture, along with its effectiveness in communicating clarity when making arguments, extends beyond its visual properties, emphasizing the importance of its proprioceptive dimension. This dimension involves the physical sensations associated with performing the gesture, such as muscular tension, control power, and sharpness. The speaker experiences these proprioceptive qualities firsthand, while interlocutors empathically perceive them, making the understanding of a gesture not only a visual experience but also a physical one.

The kinesthetic sensations of sharpness, determination, and control which are qualities integral to the Slicing gesture, make it an effective semiotic resource for stance-taking and for “self-presentation” (Streeck, 2008) in political communication. These proprioceptive qualities not only reinforce the speaker’s self-perception of decisiveness and determination but also communicate these attributes to the audience. This alignment between physical sensation and communicative intent forms the basis for the habitualization and stabilization of this gesture. “(H)ow stances are taken, and which stances are taken, are often habitually repeated by people with similar identities” (Kiesling, 2022, p. 412). Thus, it is unsurprising that the Slicing gesture emerges as a prevalent gesture in the domain of political communication. “[G]estures reveal a great deal about interactional practices, the social norms that underlie them, and how local and wider ideologies in societies shape the nature of gestures and their use” (Brookes and Le Guen, 2019, p. 129). Accordingly, if certain practices, norms, and ideologies are shared among speech communities, they may share gestural forms.

## Data Availability

The dataset analyzed in this study is not publicly available; however, the data used in the analysis are included within the article. Questions regarding further information on this data should be directed to the corresponding author.

## Footnotes

^1^Personal communication.

^2^Quantitative analyses of this aspect are currently in progress.

^3^This observation is reinforced by the quantitative analysis, which shows that defining the position of others, such as a political party or journalists, is more prevalent when the Slicing gesture is performed with the palm facing the speaker’s body. This variant is often moved along the sagittal plane, as Calbris also observed.

^4^Online Etymology Dictionary, s.v. decisive (https://www.etymonline.com/search?q=decisive).

^5^One possible explanation for how interlocutors may experience a proprioceptive sense of a seen gesture lies in the concept of mirror neurons. These neurons are thought to allow observers to internally simulate observed actions, which might account for how the Slicing gesture’s qualities of power, control, and sharpness are “felt” by the interlocutor. For a broader discussion, see Gallese et al. (1996) on mirror neuron systems and their role in embodied communication.
